# Selective Uptake of Pelagic Microbial Community Members by Caribbean Reef Corals

**DOI:** 10.1128/AEM.03175-20

**Published:** 2021-04-13

**Authors:** Kenneth D. Hoadley, Maria Hamilton, Camille L. Poirier, Chang Jae Choi, Cheuk-Man Yung, Alexandra Z. Worden

**Affiliations:** a Monterey Bay Aquarium Research Institute, Moss Landing, California, USA; b GEOMAR Helmholtz Centre for Ocean Research, Kiel, Germany; University of Queensland

**Keywords:** bentho-pelagic coupling, coral heterotrophy, picoplankton

## Abstract

Coral reefs are possible sinks for microbes; however, the removal mechanisms at play are not well understood. Here, we characterize pelagic microbial groups at the CARMABI reef (Curaçao) and examine microbial consumption by three coral species: *Madracis mirabilis*, *Porites astreoides*, and *Stephanocoenia intersepta*. Flow cytometry analyses of water samples collected from a depth of 10 m identified 6 microbial groups: *Prochlorococcus*, three groups of *Synechococcus*, photosynthetic eukaryotes, and heterotrophic bacteria. Minimum growth rates (μ) for *Prochlorococcus*, all *Synechococcus* groups, and photosynthetic eukaryotes were 0.55, 0.29, and 0.45 μ day^−1^, respectively, and suggest relatively high rates of productivity despite low nutrient conditions on the reef. During a series of 5-h incubations with reef corals performed just after sunset or prior to sunrise, reductions in the abundance of photosynthetic picoeukaryotes, *Prochlorococcus* and *Synechococcus* cells, were observed. Of the three *Synechococcus* groups, one decreased significantly during incubations with each coral and the other two only with *M. mirabilis.* Removal of carbon from the water column is based on coral consumption rates of phytoplankton and averaged between 138 ng h^−1^ and 387 ng h^−1^, depending on the coral species. A lack of coral-dependent reduction in heterotrophic bacteria, differences in *Synechococcus* reductions, and diurnal variation in reductions of *Synechococcus* and *Prochlorococcus*, coinciding with peak cell division, point to selective feeding by corals. Our study indicates that bentho-pelagic coupling via selective grazing of microbial groups influences carbon flow and supports heterogeneity of microbial communities overlying coral reefs.

**IMPORTANCE** We identify interactions between coral grazing behavior and the growth rates and cell abundances of pelagic microbial groups found surrounding a Caribbean reef. During incubation experiments with three reef corals, reductions in microbial cell abundance differed according to coral species and suggest specific coral or microbial mechanisms are at play. Peaks in removal rates of *Prochlorococcus* and *Synechococcus* cyanobacteria appear highest during postsunset incubations and coincide with microbial cell division. Grazing rates and effort vary across coral species and picoplankton groups, possibly influencing overall microbial composition and abundance over coral reefs. For reef corals, use of such a numerically abundant source of nutrition may be advantageous, especially under environmentally stressful conditions when symbioses with dinoflagellate algae break down.

## Abstract (precis)

We identify interactions between coral grazing behavior and the growth rates and cell abundances of pelagic microbial groups found surrounding a Caribbean reef. During incubation experiments with three reef corals, reductions in microbial cell abundance differed according to coral species and suggest specific coral or microbial mechanisms are at play.

## INTRODUCTION

Recent interest in the complex biospheres found within coral colonies has renewed attention to the microbial diversity found on tropical reefs (1, 2). For example, the composition of bacterial communities associated with coral (i.e., the microbiome) can be quite different across coral species and/or individual colonies (3). Even eukaryotic protists living in symbiosis (Symbiodiniaceae and corallicolids) or within the calcium carbonate skeleton (endolithic microalgae) can differ widely across coral species and environmental conditions (4, 5). While symbioses between reef corals and their symbiotic algae are well studied, the significance of interactions between free-living microbial communities overlying reefs and reef corals themselves are much less well characterized.

The microbial communities found in the water column surrounding and overlying coral reef systems are largely comprised of small phytoplankton such as picoeukaryotes, *Prochlorococcus*, *Synechococcus*, and heterotrophic bacteria (6–10). Reductions in particulate organic matter (POM), as measured by carbon isotope ratios and carbon-to-nitrogen ratios, suggest that water column phytoplankton concentrations decrease across the reef crest in fringing reef systems (11). Indeed, flow cytometry-based quantification in an Australian fringing reef system with directional wave motion across the reef to the lagoon showed that concentrations of the cyanobacteria, *Prochlorococcus* and *Synechococcus*, small photosynthetic eukaryotes, and heterotrophic bacteria are higher over reef zones than in the sandy-bottomed lagoon behind the reef (shoreside) (12). This raises questions about whether or not reef systems can remove pelagic microbial cells from the overlaying water column and, if so, by what mechanism and to what extent (11–13). Similar types of bentho-pelagic coupling have been well studied for sponges (14, 15). These invertebrates filter both living and nonliving particulate (detrital) and dissolved organic matter (DOM) from the surrounding water column (16–18). Studies further indicate preferential selection of living microbial particles (7). Furthermore, sponge-pelagic coupling has been shown to influence microbial community structure. On the southwestern coast of Australia, the demosponge *Callyspongia* was reported to preferentially filter out *Synechococcus* and flow cytometry-identified high-DNA-density bacteria over low-DNA-density bacteria, changing percentages of these microbial groups within the outgoing water flow (7, 19). In the Florida Keys, the giant barrel sponge *Xetospongia muta* was shown to preferentially consume *Prochlorococcus* and *Synechococcus* over picoeukaryotes and bacteria, with preferential selection of the latter two occurring in a concentration-dependent manner (higher selectivity at higher concentrations) (7). Thus, these reef invertebrates can alter the relative abundance of different pelagic microbial groups in the outgoing water that is returned to the broader system. Microbial communities are regulated by both bottom-up (nutrient availability) and top-down (predation and viral infection) processes within the marine environment (20). Selective feeding behaviors by reef invertebrates may serve as an important top-down regulatory mechanism that influences heterogeneity and diversity in the surrounding microbial community.

Like sponges, corals are also capable of filter feeding, but most studies have focused on capture and predation of relatively large (>100-μm) planktonic organisms (21). However, using cultured eukaryotic algae as prey or concentrated seawater, a few studies have shown that the consumption of photosynthetic eukaryotes, cyanobacteria, and heterotrophic bacteria may also serve as a source of nutrition for reef corals (22–24). This numerically abundant source of food could be important for overall coral nutrition, supplementing the supply of energy-rich photosynthate translocated to the coral from symbiotic algae (Zooxanthellae) living within the host corals’ gastrodermal cells. Under stressful conditions, the host coral interaction with these symbiotic algae can be disrupted such that greater reliance on heterotrophy is required to meet coral metabolic demands (25). Hence, selective feeding on microbial cells may be particularly important for corals under environmentally stressful conditions when demand for heterotrophically derived carbon is high (26).

For feeding corals, individual polyps extend tentacles to capture prey, which are then retracted and bring prey to the mouth for consumption (21). Other coral behaviors, such as the excretion of a mucus layer, may also entrap planktonic cells and/or detritus. Depending on the species, this mucus layer is then consumed by the coral (21, 27, 28) or released into the water column (14, 28, 29), thereby removing bacteria and/or particulate material. It is unclear how or if tentacle capture or the mucus layer elicits any selective mechanism for preferentially feeding upon certain groups of picoplankton. Nevertheless, recent studies that have isotopically labeled (^15^N) the pelagic microbial community have demonstrated that fixed N is indeed incorporated into the host coral and Zooxanthellae alga through the capture and consumption of diazotrophs (26, 30, 31). Prior studies have also indicated that some coral species can shape microbial community composition on the reef (6, 14, 32). However, differences in methodologies, systems investigated, and both coral and microbial community members examined leave gaps in our understanding of bentho-pelagic coupling involving direct predation by corals.

In this study, we characterized growth rates, cell abundance, and species composition of the microbial community within the water column at a reef site along the southern coast of Curaçao. Using incubation experiments, we then characterized predation of this microbial community by three coral species (*Madracis mirabilis*, *Porites astreoides*, and *Stephanocoenia intersepta*) commonly found throughout the Caribbean Sea (33). Incubation experiments were conducted in the early and late evening to assess temporal differences in coral predation. Our study shows that corals exhibit strong selective preferences for specific microbial groups in small size fractions and that rates of coral predation appear to be linked to diurnal aspects of picoplankton biology and physiology.

## RESULTS

**FIG 1:**
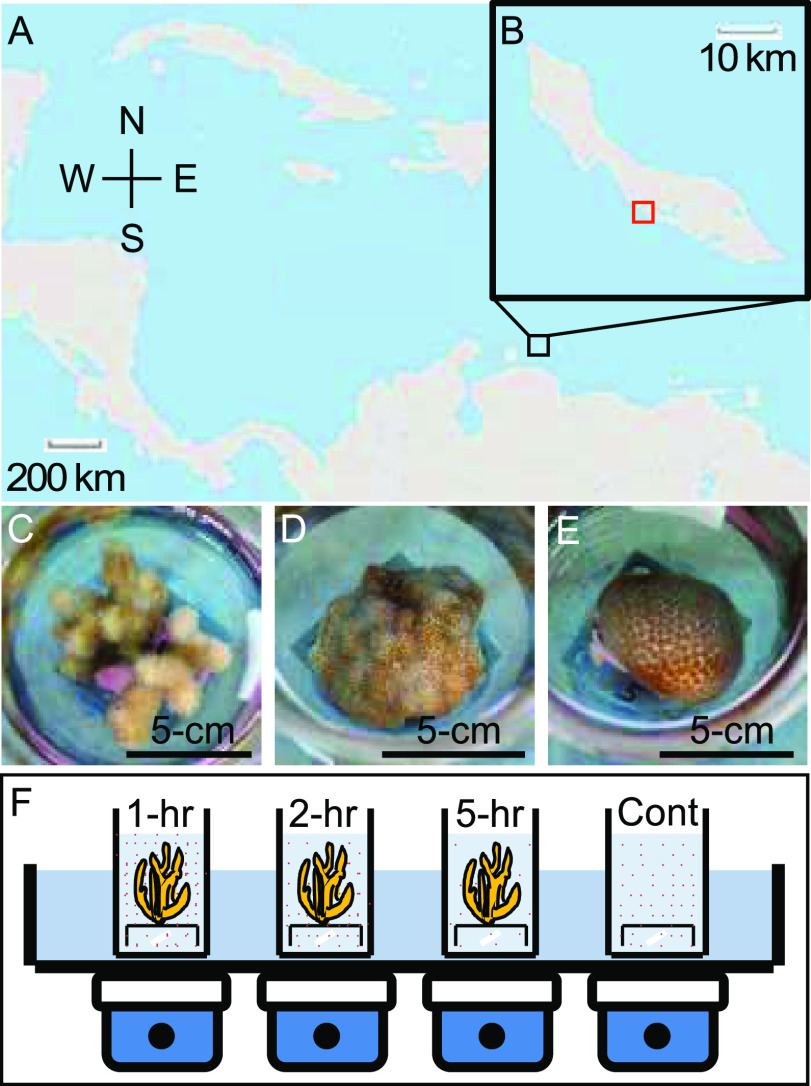
Site map and representative fragments from the corals investigated. (a and b) Research was carried out on the southern coast of Curaçao, at the CARMABI research institute. (Maps were created with imapbuilder.net.) Panels c to e reflect the three coral species utilized in this study (*M. mirabilis*, *P. astreoides*, and *S. intersepta*, respectively). Images were taken while corals were mounted on PVC tiles and sitting in incubation chambers. (f) The cartoon figure is representative of the clearance rate chambers utilized in the experimental design.

**FIG 2:**
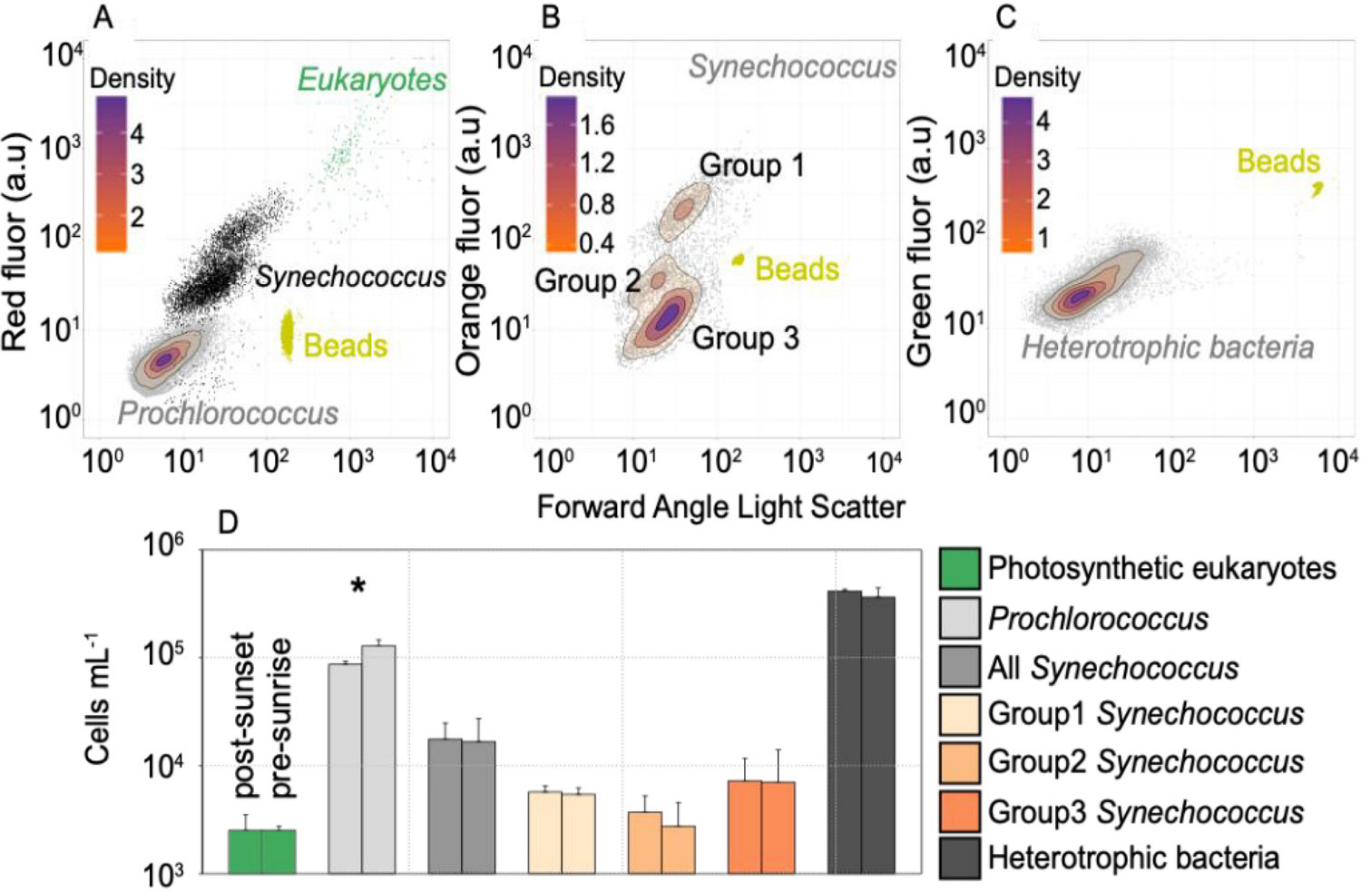
Abundance of pelagic microbial groups detected by flow cytometry. Panels a to c are from a single initial postsunset bulk water sample and represent the six microbial populations evaluated in this study. Only populations derived from final gated windows are shown in each cytogram panel. YG fluorescent beads (0.75 μm) are represented in each graph (yellow). To help visualize count density, certain populations are topographically overlaid within each graph. (a) The *Prochlorococcus* (topograph) group, along with small photosynthetic eukaryotes (green dots), are observed in the cytogram. (b and c) The three *Synechococcus* groups are reflected in the cytogram (b), and SYBR green-stained bacteria are shown (c). (d) Bar graphs show the average abundance (on a logarithmic scale) of each microbial group at the initiation of the postsunset and presunrise incubations (*n* = 4). Microbial groups with significant differences in cell abundance between incubation periods are noted with an asterisk (*, *P* <0.05).

In all, six microbial groups were distinguished by flow cytometry from water samples collected throughout experimental procedures at the CARMABI reef site on the southern coast of Curaçao (Fig. 1). The photosynthetic community was comprised of small eukaryotes (Fig. 2a), *Prochlorococcus* (Fig. 2a), and three groups of *Synechococcus* (Fig. 2b). The abundance of heterotrophic bacteria (Fig. 2c) was also quantified. All data are from a single site, and standard deviations reflect replicate (*n* = 4) samples from the same time point (postsunset or presunrise). The average abundance of photosynthetic eukaryotes was similar at the onset of night (postsunset, 2,539 ± 971 cells ml^−1^) and 5.5 h after sunset (2,526 ± 232 cells ml^−1^) (Fig. 2d). *Prochlorococcus* abundance increased significantly (*t* test, *P = *0.041) from 87,113 ± 6,108 cells ml^−1^ (postsunset) to 129,375 ± 17,253 cells ml^−1^ (5.5 h after sunset). Average cell abundances for all *Synechococcus* were also similar between postsunset (17,711 ± 7,143 cells ml^−1^) and 5.5 h after sunset (16,783 ± 10,472 cells ml^−1^). High variability within the *Synechococcus* population largely stems from group 3, where abundances differed considerably between the first (12,008 ± 1,178 cells ml^−1^) and second (2,223 ± 1,403 cells ml^−1^) evenings of incubation. Lastly, heterotrophic bacterial cell abundances averaged 416,747 ± 15,026 cells ml^−1^ and 365,784 ± 79,375 cells ml^−1^ at the start of postsunset and 5.5 h after sunset.

### Microbial community dynamics, growth rates, and diversity.

From cell concentrations derived from control samples, we computed minimum growth rates of the different microbial groups (Table 1), several of which increased more during postsunset incubations than in presunrise incubations. These results are consistent with known aspects of timing in phytoplankton cell division (34–36) and resulted in higher growth rates in the postsunset period (Table 1). This was observed for *Prochlorococcus*, which also exhibited significantly greater mean forward angle light scatter (FALS; a proxy for cell size) in the postsunset period, *Synechococcus* group 1, and *Synechococcus* group 3 (Table 2). Comparisons of FALS and fluorescence measurements between *T*_0_ and *T*_5_ time points were also performed and indicate changes in cell physiology that could be captured by flow cytometry occurring during the postsunset incubation, consistent with this period being when the bulk of cyanobacterial cell division occurs (see Tables S3 and S4 in the supplemental material) (37–40).

**TABLE 1:** Growth rates and cell physiological metrics (average ± SD, *n* = 4) for microbial groups during early and late incubation periods, as calculated from controls^*a*^

Microbial group	Growth rate (μ) 5 h^−1^		*P* value	Minimum growthrate (μ) day^−1^
	Postsunset incubation	Presunrise incubation		
Photosynthetic eukaryotes	0.22 ± 0.16	0.23 ± 0.10	0.9321	0.45
*Prochlorococcus*	**0.47 ± 0.09**	**0.08 ± 0.02**	0.0023	0.55
*Synechococcus* group 1	**0.44 ± 0.05**	**0.07 ± 0.05**	0.0001	0.51
*Synechococcus* group 2	0.25 ± 0.17	0.01 ± 0.06	0.0559	0.26
*Synechococcus* group 3	**0.16 ± 0.11**	**−0.05 ± 0.03**	0.0259	0.11
Heterotrophic bacteria	0.06 ± 0.08	−0.09 ± 0.27	0.4465	NA

a The minimum daily growth rate is taken as the sum of the two measured 5-h periods. Boldface values reflect significant differences (*t* test, *P* value shown) across the postdusk and predawn incubation periods. Note that the minimum daily growth rate does not include growth that was removed via protistan predation and, hence, likely would be higher if protistan predators were not active during our experiments (not tested). NA, not available. Heterotrophic growth rates were near or below zero in our experiments; hence, we did not estimate a minimum daily rate.

**TABLE 2:** Average FALS and red fluorescence^*a*^

Microbial group and parameter	Value (BRU) for:		*P* value
	Postsunset incubation	Presunrise incubation	
FALS
*Prochlorococcus*	**0.45 ± 0.02**	**0.30 ± 0.01**	0.0001
*Synechococcus* group 1	**0.80 ± 0.02**	**0.70 ± 0.00**	0.0015
*Synechococcus* group 2	**0.68 ± 0.04**	**0.58 ± 0.02**	0.0060
*Synechococcus* group 3	0.69 ± 0.02	0.68 ± 0.03	0.6274
Red fluorescence
*Prochlorococcus*	0.77 ± 0.04	0.69 ± 0.08	0.8921
*Synechococcus* group 1	2.19 ± 0.07	2.02 ± 0.06	0.3468
*Synechococcus* group 2	1.75 ± 0.04	1.69 ± 0.13	0.8637
*Synechococcus* group 3	1.57 ± 0.02	1.56 ± 0.09	0.3555
Orange fluorescence
*Synechococcus* group 1	**1.40 ± 0.03**	**1.35 ± 0.07**	0.0189
*Synechococcus* group 2	0.99 ± 0.09	1.01 ± 0.13	0.5280
*Synechococcus* group 3	0.73 ± 0.09	0.80 ± 0.09	0.9372

a Average FALS and red fluorescence values are displayed in bead relative units (BRU) and were only calculated for *Prochlorococcus* and *Synechococcus* groups. FALS and red fluorescence values were not calculated for photosynthetic eukaryotes, as they reflect a much more biologically diverse assemblage, and the averages would not be informative. Average orange fluorescence (BRU) was only calculated for *Synechococcus* groups, as they contain phycoerythrin. Boldface values reflect significant differences (*t* test, *P* value shown) across incubation periods.

**FIG 3:**
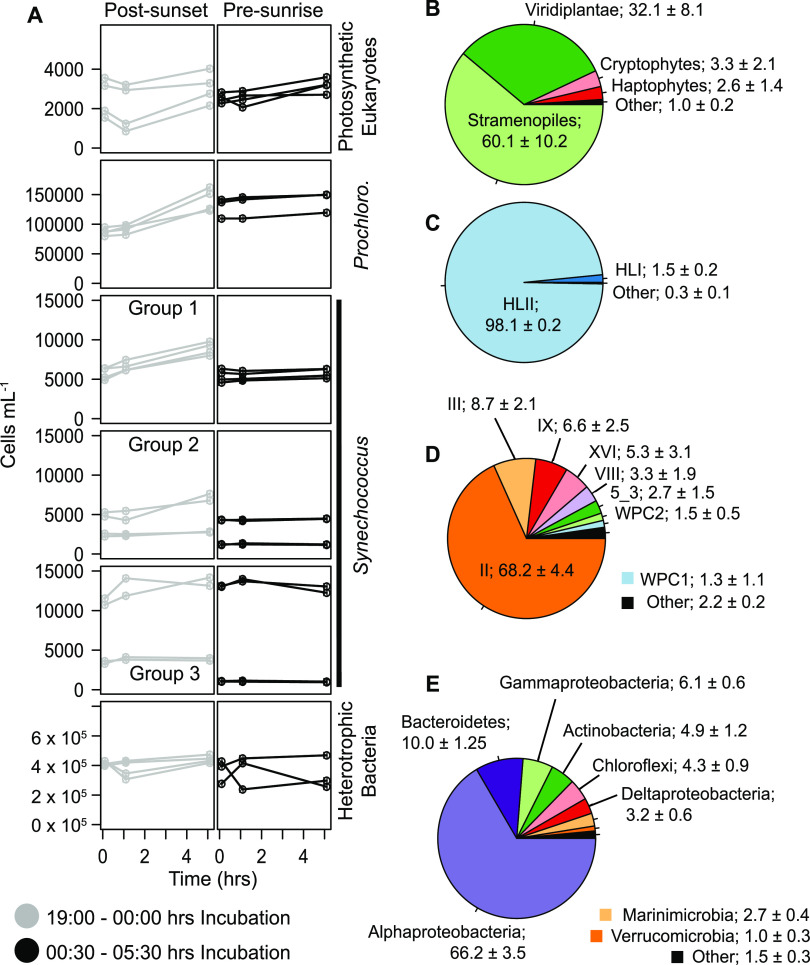
Temporal changes in microbial abundance. (a) Individual group cell counts are shown from each control incubation and sample point (0, 1, and 5 h). The two 5-h incubations each night are reflected in gray (19:00 h, postsunset) and black (01:00 h, presunrise). For each incubation experiment, V1-V2 16S rRNA gene amplicons were also analyzed from the seawater collected for distribution at *T*_0_. Shown here are the average amplicon percentages for small photosynthetic eukaryotes (b), *Prochlorococcus* (c), *Synechococcus* (d), and heterotrophic bacteria (e). Within each pie chart, groups comprising less than 1% of amplicons were grouped together and marked as other, and numbers shown reflect the average percent and SD (*n* = 4).

Community structure analyses of V1-V2 16S rRNA gene amplicon sequence variants (ASVs) provided higher taxonomic resolution of the groups identified by flow cytometry. Rarefaction curve analyses indicated comparable sampling, with the depth of sequencing being at, or approaching, saturation (Fig. S2 and Table S1). The average Shannon diversity index (*H*) and evenness (E_H_) for all 4 samples is 5.85 ± 0.13 and 0.76 ± 0.01, respectively. The largest proportion of amplicons from photosynthetic eukaryotes (plastid derived) belonged to green algae (32.1% ± 8.1%) and stramenopiles (60.1% ± 10.2%), with more minor contributions of cryptophytes and haptophytes (Fig. 3b). *Micromonas* clades A/B/C (with these clades being indistinguishable using the V1-V2 16S rRNA) comprised the largest proportion of green algal amplicons (31% ± 4%). Among stramenopile ASVs, diatoms, especially *Pseudonitzschia serrata*, Skeletonema costatum, *Actinocyclus subtilis*, and *Nitzschia thermali*, comprised the largest proportion (68% ± 12%) (Fig. 3b and supplemental material). *Prochlorococcus* amplicons were dominated by the HLII ecotype (98.1% ± 0.2%), appearing to fit with the single, low-chlorophyll-fluorescence population identified by flow cytometry (Fig. 2a and 3c). Among *Synechococcus* amplicons, clade II dominated (68.2% ± 4.4% of all *Synechococcus*) and presumably connects to flow cytometric group 3 (Fig. 2b and 3d). Other notable clades with contributions between 5 and 10% of *Synechococcus* amplicons were III, IX, and XVI. Alphaproteobacterial amplicons comprised 66% ± 3.4% of all heterotrophic bacterial sequences, with an average of 21% ± 4.7% of the total being SAR11 (mostly clade Ia, but clades II, IV, and Ib also were present), with both *Gammaproteobacteria* and *Actinobacteria* being between 5 and 10%, while all other lineages represented <5% of heterotrophic bacterial ASVs (Fig. 3e).

### Coral consumption of microbial groups.

**FIG 4:**
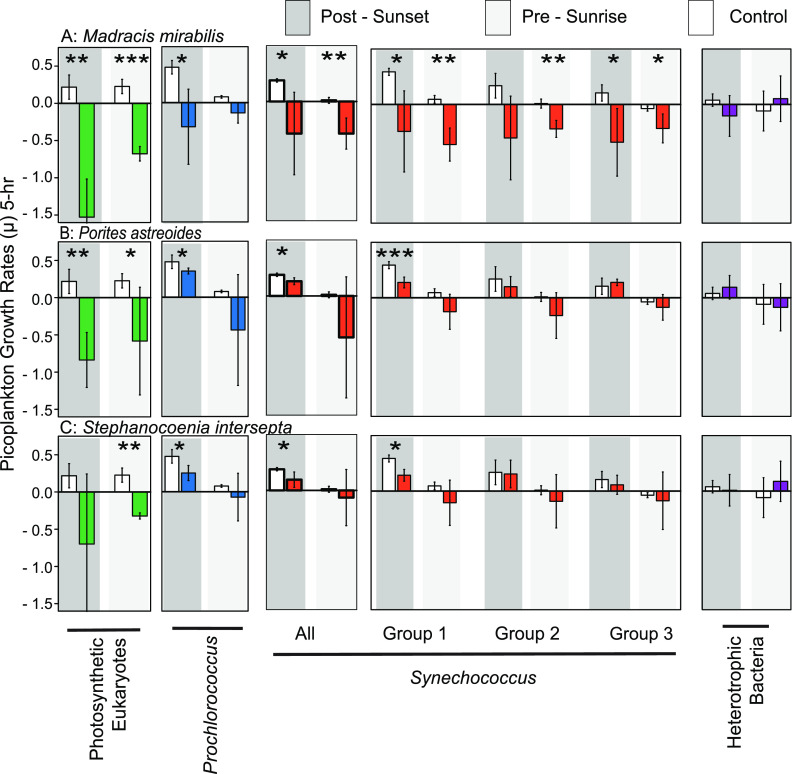
Impact of coral predation on the pelagic microbial community. Colored bars reflect growth rates [log(*T*_5_/*T*_0_)] for each picoplankton population when incubated with *M. mirabilis* (a), *P. asteroides* (b), and *S. intersepta* (c). White bars in each graph represent growth rates within control (no coral) chambers. Dark and light gray backgrounds reflect postsunset and presunrise incubation periods, respectively. All bars represent averages ± SD. For each microbial group, asterisks represent significant deviations from control samples (*, *P* < 0.05; **, *P < *0.01; ***, *P* < 0.001).

In this study, we used three coral species that are commonly found in relative health and abundance near the CARMABI reef site (Fig. 1a and b). Coral incubation data revealed that patterns of cell consumption differed across coral species and microbial plankton groups (Fig. 4). The abundance of photosynthetic eukaryotes decreased significantly when incubated with either *M. mirabilis* (*P = *0.002) or *P. astreoides* (*P = *0.005) during postsunset incubations, whereas significant decreases were observed for all three coral species during presunrise incubations (*P *values of 0.003, 0.033, and 0.002 for *M. mirabilis*, *P. astreoides*, and *S. intersepta*, respectively). Significant reductions in *Prochlorococcus* were only observed during postsunset incubations across all coral species (*P *values of 0.021, 0.025, and 0.033 for *M. mirabilis*, *P. astreoides* and *S. intersepta*, respectively). For *M. mirabilis*, *Synechococcus* groups 1 and 3 decreased in abundance over time during the postsunset incubation (*P* = 0.014 and *P* = 0.014 for groups 1 and 3, respectively), while all three groups decreased later in the evening during the presunrise incubation (*P* = 0.001, *P* = 0.004, and *P* = 0.041 for groups 1, 2, and 3, respectively). In contrast, significant decreases in group 1 abundances were observed when incubated with the coral *P. astreoides* (*P* = 0.001) or *S. intersepta* (*P* = 0.018) during postsunset incubations only. For each coral, grazing rates were also calculated for the total *Synechococcus* community. Not surprisingly, we again saw significant reductions in the full *Synechococcus* population when incubated with *M. mirabilis* under both postsunset (*P* = 0.050) and presunrise (*P* = 0.004) incubations. However, significant reductions in total *Synechococcus* cells were observed only during the postsunset incubation for *P. astreoides* (*P* = 0.050) or *S. intersepta* (*P* = 0.050). No significant reductions in bacterial abundance were observed in either postsunset (*P* values of 0.478, 0.680, and 0.835 for *M. mirabilis*, *P. astreoides* and *S. intersepta*, respectively) or presunrise (*P *values of 0.653, 0.912, and 0.442 for *M. mirabilis*, *P. astreoides* and *S. intersepta*, respectively) incubation periods.

### Carbon dynamics in the water column.

**FIG 5:**
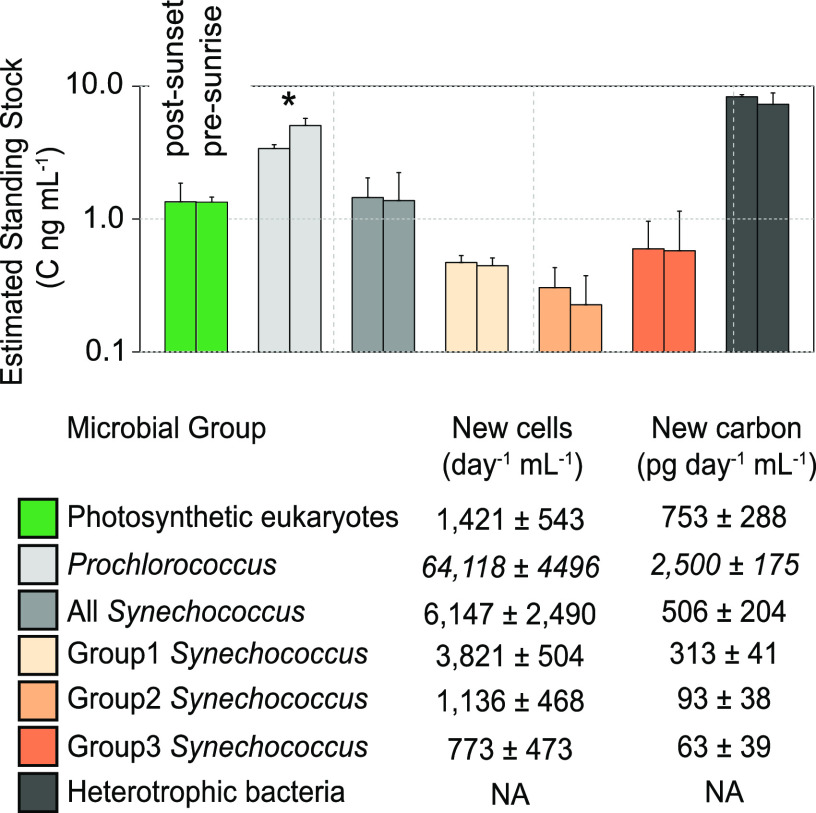
Estimated standing stocks and daily production of microbial groups overlaying the reef. (Top) Values represent estimates (on a logarithmic scale) using carbon conversion factors derived from references 41 and 42 and total cell abundances calculated from *T*_0_ time points for each incubation. Microbial groups with significant differences in cell abundance between incubation periods are denoted by an asterisk (*, *P* < 0.05). (Bottom) Minimum daily number of cells produced and estimated carbon production for each microbial group, calculated using the measured minimum daily growth rate (sum of postsunset and presunrise incubations). All values reflect the averages ± SD, with *n* = 4.

Cell abundance data allowed us to estimate the carbon biomass of each microbial group, using established conversion factors (Fig. 5) for the photosynthetic groups (41) and for heterotrophic bacteria (42). Average carbon (C) represented by the photosynthetic eukaryotes was 1.35 ± 0.51 ng C ml^−1^ at the onset of night (postsunset) and 1.34 ± 0.12 ng C ml^−1^ at 5.5 h after sunset (presunrise). *Prochlorococcus* cells represented 3.39 ± 0.24 ng C ml^−1^ at postsunset and then significantly (*t* test, *P = *0.041) increased to 5.04 ± 0.67 ng C ml^−1^ at 5.5 h after sunset. Total *Synechococcus* cells comprised 1.45 ± 0.24 ng C ml^−1^ at postsunset and 1.37 ± 0.85 ng C ml^−1^ at 5.5 h after sunset. Lastly, total carbon within the water column represented by heterotrophic bacterial cells was 8.33 ± 0.33 ng C ml^−1^ at postsunset and 7.3 ± 1.59 ng C ml^−1^ at 5.5 h after sunset (Fig. 5).

Minimum daily growth rates, calculated as the sum of the postsunset and presunrise growth rates from control (no coral feeding) samples, were utilized to determine the total daily production of carbon by each microbial group (Fig. 5). Photosynthetic eukaryotes produced 753 ± 288 pg C day^−1 ^ml^−1^, while *Prochlorococcus* and the combined *Synechococcus* groups produced 2,500 ± 175 and 506 ± 204 pg C day^−1 ^ml^−1^, respectively. Low growth rates in control incubations prohibited the calculation of net carbon production by heterotrophic bacteria. The removal of carbon from the water column by coral predation varied across species and totaled 387.9 ± 152.6 ng h^−1^, 106.8 ± 27.3 ng h^−1^, and 138.7 ± 62.2 ng h^−1^ for the corals *M. madracis*, *P. astreoides*, and *S. intersepta*, respectively (Table S5). *Prochlorococcus* represented the majority of removed carbon via predation (215.7 ± 103.6 ng h^−1^, 42.8 ± 35.6 ng h^−1^, and 94.7 ± 54.5 ng h^−1^), followed by photosynthetic eukaryotes (101.6 ± 18.2 ng h^−1^, 65.5 ± 6.9 ng h^−1^, and 47.5 ± 38.5 ng h^−1^) and, lastly, *Synechococcus* (70.7 ± 58.7 ng h^−1^, 9.2 ± 4.7 ng h^−1^, and 20.1 ± 12.2 ng h^−1^) for the corals *M. madracis*, *P. astreoides*, and *S. intersepta* (Table S3). Importantly, calculations were generated from postsunset incubations and should be considered peak rates of carbon removal, as corals are considered nocturnal and grazing rates are highest in the evening (43, 44). Also, while colony surface area was not directly measured, all colonies were approximately 25 cm^2^, and carbon removal rates reflect the average across colonies within each coral species.

## DISCUSSION

The abundance of different groups within the pelagic microbial community overlying reefs has been studied in both Pacific and Caribbean coral reef ecosystems (6, 7, 10, 45, 46). Some of these studies have also addressed the removal of various microbial groups by reef organisms (6, 7), addressing important mechanisms that drive bentho-pelagic interactions on coral reefs. Likewise, amplicon-based approaches have been widely used to investigate the coral holobiont (2) and, to a lesser extent, to describe the diversity of the pelagic microbial community surrounding coral reefs (10, 46). However, few studies have addressed how interactions between cellular abundance, growth rates, diversity, and predation impact planktonic microbial groups near coral reefs. Here, we identify interactions between corals and microbial groups, namely, selective predation by corals, that influence the composition of different groups within pelagic microbial communities overlaying reef ecosystems (2, 46).

For oligotrophic waters surrounding coral reef systems, phytoplankton groups within the water column are of particular interest due to their relatively high abundance and role as primary producers. Our results show that at the CARMABI reef site, the abundance of *Prochlorococcus*, *Synechococcus*, and photosynthetic eukaryotes was, on average, 108,244 cells ml^−1^, 17,247 cell ml^−1^, and 2,533 cells ml^−1^, respectively (Fig. 2d). Abundances are similar to those observed in deeper water reef sites along the Florida Reef Tract (7) or offshore along the southern coast of Cuba (10). The CARMABI reef site is located on the southern coast of Curaçao, facing South America, but separated from that land mass by a deep-water trench (1,000 m). The steep gradient into deep waters along the southern coast likely influences the microbial community, potentially by minimizing the influence of anthropogenic nutrient inputs and runoff within the water column. Thus, the microbial community at the CARMABI reef is more reflective of deeper water sites despite the relatively shallow (10 m) depth from which samples were taken.

While broad microbial plankton groups can be classified using flow cytometry, coupling this method with an examination of community composition based on analysis of 16S rRNA gene amplicons provides even greater resolution. The HLII clade was dominant within *Prochlorococcus* amplicon reads from all water samples in this study (98%) (Fig. 3c). This is in contrast to results thus far from northern Caribbean reefs in the Florida Reef Tract or island chains off the southern coast of Cuba, in which the LLIV clade appears to dominate (10). For the CARMABI reef, the majority of *Synechococcus* amplicons were represented by clade II (68%) (Fig. 3d), which is typically present in warmer coastal/continental shelf waters at low latitudes (47). The clade III strain was also notable (8.7%) within our sampling and is commonly found in oligotrophic environments (47, 48). Interestingly, three distinct cytometry-based phenotypes are observed for *Synechococcus* and differ in terms of orange fluorescence, suggesting various cellular concentrations of phycoerythrin across the groups (Fig. 2b). While type II and III were the most abundant *Synechococcus* genotypes observed (Fig. 3d), neither could be reliably attributed to a cytometry-based phenotype. Hence, other approaches will be needed to directly connect these genotypes to cytometry-based populations.

Among amplicons from eukaryotic phytoplankton, stramenopiles and green algae were dominant (Fig. 3b). Diatoms comprised 68% of all stramenopile amplicons, but as a fraction of all phytoplankton amplicons, they comprise only 4% of the total at the CARMABI reef. Among the green algae, the *Micromonas* clade/A/B/C represented by *M. commoda* was most prevalent, with *Ostreococcus* and *Mantoniella* also being observed. Class II prasinophytes, which include the abovementioned *Micromonas* and *Ostreococcus* groups, are known to be broadly distributed throughout the world’s oceans (49); thus, it is not surprising that they are also found within the water column above coral reef systems. Overall, the free-living phytoplankton community overlaying the CARMABI reef system represents a genetically diverse and numerically abundant assemblage with high rates of productivity that may represent an important source of nutrition for filter-feeding invertebrates.

Cell division in *Prochlorococcus* (37, 38, 50) and *Synechococcus* (39, 40) has been shown to often start just after sunset, as observed here. This resulted in the higher growth rates and significant reductions in FALS and fluorescence values recorded during postsunset incubations (Table 1; see also Tables S3 and S4 in the supplemental material). For *Prochlorococcus*, the high division rates early in the evening resulted in higher cell abundance during the presunrise incubation period, similar to diel patterns of cyanobacterial cell abundance observed across reef sites in the U.S. Virgin Islands (8, 9). However, significant reductions in *Prochlorococcus* cell abundance due to predation by all three corals only occurs during the postsunset incubation period, suggesting that predation is not driven by prey availability alone (Fig. 4a to c). Similarly, for *Synechococcus*, predation by the corals *P. astreoides* and *S. intersepta* only occurs during peak cell division rates and is largely driven by preferential consumption of *Synechococcus* group 1, where growth rates are highest (Fig. 4b and c, Table 1). Although we are unaware of *Synechococcus* group-specific data, predation rates by heterotrophic nanoflagellates (HNF) in the northwest Mediterranean Sea are not correlated with the abundance of *Synechococcus* (51), which they have been observed to consume. However, the number of *Synechococcus* cells found within the food vacuoles of HNF was inversely related to the number of *Synechococcus* undergoing cellular division. The timing of predatory activity is likely driven by aspects of prey cellular physiology, including cell size, which is thought to be a limiting factor for HNF cells. Similarly, differential selection of microbial groups (for consumption) by individual coral species may also favor cells undergoing cellular division, thereby maximizing their nutritional value. Importantly, other aspects of cyanobacterial cell biology vary on a diurnal basis beyond size. For the cyanobacterium *Synechococcus* sp. strain PCC 6803, glycogen and ATP reserves are highest just after sunset and decrease throughout the night as metabolic requirements are met through the consumption of stored energy (39, 40, 52). Glycogen is the main compound translocated by symbiotic dinoflagellates (Symbiodiniaceae) and can provide energy for up to 80% of the host’s metabolic needs (53). Therefore, coral behavior may favor higher grazing rates during the early evening, when the nutritional value of each *Prochlorococcus* or *Synechococcus* cell is maximized. Additionally, growth rates and cellular glycogen content are directly proportional within *Synechococcus* sp. strain PCC 6803 (54). In our study, *Synechococcus* group 1 had a higher growth rate than the other two groups (Fig. 4). If growth rates and glycogen content are similarly correlated within our study, then differences in growth rates across our three *Synechococcus* groups also indicate nutritional differences, potentially explaining the preferential selection observed here for group 1 (Fig. 2b, Table 1) by the corals *P. astreoides* and *S*. *intersepta*. The Mediterranean coral *Stylophora pistillata* preferentially fed on *Synechococcus* over *Prochlorococcus* under high-temperature conditions, when supplemental nutrients are beneficial toward mitigating thermal stress (26). This form of preferential selection is thought to occur due to the higher nitrogen content in *Synechococcus* than *Prochlorococcus*, further supporting the potential importance of grazing selection by reef corals.

While *P. astreoides* and *S. intersepta* displayed a strong temporal pattern of predation, the coral *M. mirabilis* consumed a significant proportion of *Synechococcus* cells during both postsunset and presunrise incubation periods (Fig. 4a). Furthermore, *M. mirabilis* appears to be far less selective in terms of which *Synechococcus* group it consumed (Fig. 4a). Generally speaking, most corals extend their tentacles at night for feeding (55–57). However, grazing behaviors can differ across coral species, largely depending on their reliance on heterotrophy versus autotrophy under given environmental conditions (25, 58). Importantly, the proportion of photosynthate translocated from the Zooxanthellate algae to the host coral can differ substantially across species or environmental conditions (59–61). While not identified here, previous Caribbean-based work shows the Zooxanthellae *Symbiodinium* A3 species to be associated with *S. intersepta*, and the *Symbiodinium* A4a species is likely a dominant strain within *P. astreoides*, although *Breviolum* and *Cladocopium* symbionts also can be found in *P. astreoides* (62, 63). For *M. mirabilis* corals in Curaçao, a *Breviolum* species symbiont was the only type identified through sequencing of the rRNA gene large subunit (64). Zooxanthellae species within these corals may also vary in their contribution of photosynthate to the host, potentially driving the corals’ overall reliance on heterotrophy versus autotrophy. Furthermore, under optimal (nonstress) conditions, corals that rely heavily on autotrophy may view the energy expenditure for feeding on picoplankton as only favorable when the nutritional value of the prey is highest. In contrast, species that rely more heavily on heterotrophy may be more inclined to feed throughout the night, regardless of the nutritional quality of the picoplankton prey. Differences in lifestyle strategies may help explain the differences in *Prochlorococcus* and *Synechococcus* predation patterns observed across coral species within this study. However, predation patterns on photosynthetic eukaryotes are more nuanced and may need to be explained through other means.

The observed clearance of eukaryotic phytoplankton from the water column agrees with previous studies on corals that utilized concentrated seawater samples or cultured phytoplankton to measure grazing rates (23, 24, 32, 65, 66). Importantly, and unlike results for the cyanobacterial groups, no clear pattern of temporal predation on photosynthetic eukaryotes arising from our three coral species as significant reductions in cell abundance are observed during both incubation periods for the corals *M. mirabilis* and *P. astreoides*, whereas for the coral *S. intersepta*, reductions are only observed during the presunrise incubation (Fig. 4). On a per-cell basis, photosynthetic eukaryotes can represent a more nutritionally valuable prey source than *Prochlorococcus* and *Synechococcus* cells (41). For corals, it may always be energetically favorable to prey on these items and not simply when their nutritional value is greatest. Alternatively, predation patterns may reflect lower concentrations or the lack of a synchronous cell cycle across all photosynthetic eukaryotes, requiring a temporally broad predation effort. Both green algae (34) and diatoms (35, 36) have well-defined patterns of cellular division. However, peak rates of cell division may occur at various times throughout the 24-h day. Lack of synchronization with respect to cell division may help explain why growth rates in controls did not vary significantly between the two incubation periods (Fig. 3a and 4). The flow cytometric group identified as photosynthetic eukaryotes harbors considerable species diversity that would preclude the observation of species-level synchronization in cellular division. Similarly, the broad diversity reflected in the heterotrophic bacterial group may also obscure the accurate determination of growth rates.

While rates of cellular division are observed for photosynthetic eukaryotes, *Prochlorococcus*, and *Synechococcus*, little change in heterotrophic bacterial cell abundance occurs during either incubation period (Fig. 3a and 4). However, our incubations focused on nighttime changes in cellular abundance, and these patterns may fundamentally differ in heterotrophic bacteria from that observed in photosynthetic cells. For example, high temporal sampling of metatranscriptomic data in the open ocean identified significant differences in peak daily expression patterns for key metabolic pathways between various heterotrophic bacterial strains and *Prochlorococcus* (67). These differences in diel transcriptomic activity suggest that heterotrophic cellular division occurs earlier in the day and simply was not caught by our evening incubation experiments. For heterotrophic bacterial capture, our results differ from those of reference 6, where significant reductions in specific bacterial species abundances were noted for *P. astreoides* in Bermuda over longer time periods (several days to weeks). However, while their analysis focuses on specific bacterial groupings, our analysis calculates changes over the total heterotrophic bacterial community and may mask nuanced selection for specific groups. Additionally, bacterial cell abundances were reportedly much higher during the experimental period at the Bermudian site (over 1,000,000 cells ml^−1^), where nitrate levels were higher (∼1 to 1.5 μM) and temperature lower (25.8°C) than those of the CARMABI site, where bacterial concentrations were much lower (average, 391,264 cells ml^−1^). Thus, significant grazing on bacteria within Bermudian waters may reflect higher encounter rates. Heterotrophic bacterial ingestion was also noted for the Mediterranean coral *Corallium rubrum*, but capture efficiencies were much lower than those for larger eukaryotic cells and also point toward preferential selection for larger picoplankton cells (66). This is in contrast to sponges, where preferential selection in favor of smaller heterotrophic bacteria is often observed (7, 19), although the extent to which the grazing habits of corals and exclusively heterotrophic sponges can be compared is unclear.

Along with prior studies that trace the consumption of ^15^N-labeled microbial cells by reef corals (26, 30, 31), our study provides a compelling argument for the importance of the pelagic microbial community as a source of nutrition for reef corals. While the temporal differences in rates of consumption or selective preference have been largely attributed to grazing behaviors by the coral (22, 23, 26), microbial cell physiology also may play a role. Certain by-products extruded from microbial cells are known to have sticky properties critical for forming cellular aggregates and biofilms within various aquatic environments (68). For example, transparent exopolymer particles (TEP) are produced by numerous marine microbes and form sticky organic matrices that promote particulate aggregation, helping to export organic carbon to ocean depth (69, 70). TEP is known to be produced by *Synechococcus* (71, 72) and *Prochlorococcus* (73), potentially increasing cellular adherence to the coral mucus layer. Production of TEP during key cellular stages in cyanobacteria could influence the patterns of cell removal from the water column observed during postsunset and presunrise incubations in this study. Importantly, such a mechanism would be independent of coral behavior. More advanced tools for directly visualizing coral-microbe interactions are needed to understand if these nuanced patterns of consumption are coral or microbially driven.

Our results suggest that the microbial community composition at the CARMABI reef site reflects the influence of the deep-water trench that runs adjacent to the southern coast of Curaçao. Daily minimum carbon production estimates at our site suggest that turnover rates, which are calculated as production over biomass (74), are greater than 0.25 day^−1^ for the total microbial community (Fig. 5). As a comparison, ^14^C isotopic pulse-labeling assays on phytoplankton indicated that total carbon turnover rates can vary between 0 and 0.3 across the central Atlantic Ocean (75). Thus, turnover rates are fairly high despite relatively low nutrient concentrations within the water column. Thus, the relatively high cellular abundances and production rates at the time of our experiments may reflect rapid turnover of carbon into higher trophic levels. Indeed, we observed not only notable predation by benthic invertebrates but also selective removal of microbial groups from the water column. Moreover, maximal rates of feeding within the corals *P. astreoides* and *S. intersepta* coincided with cellular division across *Prochlorococcus* and group 1 *Synechococcus* populations. Whether these patterns of microbial removal are driven by coral behavior or microbial cellular properties, the resulting diurnal grazing may help maximize the nutritional value of captured picoplankton cells. Such a numerically abundant source of nutrition may be of considerable advantage to reef corals, especially under environmentally stressful conditions, when symbiosis with dinoflagellate algae (Symbiodiniaceae) breaks down (26).

## MATERIALS AND METHODS

### Water and coral collection.

The study was conducted in Curaçao using corals and microbial communities collected near the CARMABI reef site (12°07′15.1′′ N, 68°58′11.6′′ W) (Fig. 1a). Just prior to the start of each incubation (see below), representative flow cytometry, nutrient, and DNA samples were collected from bulk seawater (collected through an intake pipe at a depth of 10 m) utilized to run each incubation. Samples for flow cytometry were preserved with glutaraldehyde (20 min in the dark, 0.25% final concentration; Electron Microscopy Sciences) and flash frozen (76). Unfiltered, 20-ml samples were frozen at −80°C for nutrient analysis. DNA samples were obtained by filtering biomass from 500 ml of natural seawater onto a 0.2-μm Supor filter (Pall, USA). These samples were frozen and stored at −80°C (DNA and flow cytometry) or −20°C (nutrients) until analysis. For both acclimation and experiments with corals, flowthrough seawater was supplied through an intake pipe originating at 10-m depth on the same reef site as the corals were collected. Acclimation tanks (75 liters) were kept under shaded natural lighting (midday peak of 200 μmol quanta·m^−2^·s^−1^) and seawater flowthrough rates of 50 liters h^−1^ at a water temperature of 28°C. Additional water movement was provided within each aquarium using a Hydro Koralia water pump (3,000 liters h^−1^), producing a more natural water flow for all coral fragments. Fragments from the species *M. mirabilis*, *P. astreoides*, and *S. intersepta* were collected at depths between 5 and 10 m, trimmed to fit onto 5- by 5-cm PVC tiles, and mounted using a coral-safe epoxy (Marineland Inc., USA). Corals were then placed in acclimation tanks for 11 days to recover. After 11 days of acclimation and recovery, all corals appeared healthy, with no tissue loss or consistent polyp retraction.

### Incubation experimental setup.

A total of four incubation experiments were conducted using a flowthrough water bath to maintain ambient reef temperatures (27 to 28°C). Glass beakers (500 ml) were filled with 400 ml of natural seawater, and one coral fragment was added to each except the controls, with a small stir bar providing water movement. Incubations were conducted for 5 h, with 3-ml water samples for flow cytometry taken from each beaker at 0, 1, and 5 h. Two 5-h incubation experiments were performed on each of two consecutive nights, one starting 1 h after sunset (18:00 local time) and the other 1 h after midnight (i.e., starting 5 h before sunrise). Two colonies were used per coral species in each of the four incubation experiments. Two additional beakers in each experiment served as controls with no coral colonies added; these were used to account for changes in cell density due to division or forces of mortality within the unfiltered natural seawater in the absence of corals (Fig. 1f). Only one control incubation was available during the presunrise incubation on night 1.

### Nutrient and flow cytometry analyses.

Nutrient samples were run on an autoanalyzer according to methods previously described (77). Flow cytometry samples were processed on an Influx (BD Biosciences, USA) equipped with a 488-nm, 200-mW laser and a 70-μm nozzle, using 0.2-μm-filtered 1× PBS as the sheath fluid. For photosynthetic eukaryotes, *Prochlorococcus*, and *Synechococcus*, data collection was triggered on forward-angle light scatter (FALS), while red (692/40-nm bandpass filter) and orange (572/27-nm bandpass filter) autofluorescence reflected chlorophyll and phycoerythrin, respectively, and was utilized to gate different groups, as in prior studies (76). Fluorescent polystyrene beads (0.75 μm, yellow-green; Polysciences, Inc.) were added to each sample just prior to the run (24 μl min^−1^ flow rate), and the total volume was measured as the change in weight before and after each recording. For heterotrophic bacterial counts, samples were stained with SYBR green I (ThermoFisher, USA) at 0.5% (final concentration) and incubated in the dark for 15 min. Fluorescent beads were then added to SYBR-stained samples and data collected with the trigger on green fluorescence (520/35-nm bandpass filter).

Resulting .fcs files from flow cytometry measurements were analyzed in R using the flowVIZ (78) and flowCORE (79) packages. Photosynthetic picoeukaryotes and *Prochlorococcus* cells were quantified using two separate predefined analysis windows of FALS and red fluorescence (chlorophyll). *Synechococcus* cells were consistently observed as three distinct groups throughout our samples, and each group was individually counted using an elliptical gate based on orange (phycoerythrin) fluorescence and FALS. A fourth gate encompassed all *Synechococcus* cells and was also used to remove overlapping *Synechococcus* cells from the picoeukaryote analysis window, where overlap often occurs (80). SYBR-stained heterotrophic bacterial cells were enumerated using a predefined window based on FALS and green fluorescence. SYBR-stained *Prochlorococcus* and *Synechococcus* cells also overlapped in this window and later were subtracted from the total number of enumerated cells using quantities derived from nonstained samples (as described above). FALS and red fluorescence means were normalized using YG beads for *Prochlorococcus* and *Synechococcus* and orange fluorescence for *Synechococcus*.

### DNA extraction, PCR, and sequencing.

DNA samples from seawater collected at the start of each incubation period were extracted using the Qiagen DNeasy plant kit (Qiagen, USA), with modifications, including a bead-beating step (81). DNA was PCR amplified using the primers 27FB (82) and 338RPL (83), designed to target the V1-V2 hypervariable region of the 16S rRNA gene within bacteria (with Illumina adapters) under conditions and with quality control as detailed previously (84). Briefly, 50-μl PCRs were set up with 5 μl of 10× buffer, 1 U of HiFi-Taq, 5 ng of template DNA, and 200 nM each primer. PCR cycling parameters were 94°C for 2 min, 30 cycles of 94°C for 15 s, 55°C for 30 s, and 68°C for 1 min, and a final elongation step at 68°C for 7 min. Paired-end library sequencing (2× 300 bp) was performed using the Illumina MiSeq platform.

### 16S rRNA gene amplicon analyses.

Sequences were demultiplexed and assigned to corresponding samples using CASAVA (Illumina). A 10-bp running window was utilized to trim low-quality sequence ends at a Phred quality of 25 using Sickle 1.33 (85). Paired-end reads were merged using USEARCH v10.0.240 (86) when reads had a ≥50-bp overlap maximum 5% mismatch. The merged reads were then filtered to remove reads with a maximum error rate of >0.001 or shorter than 200 bp. Only sequences with exact matches to both primers were kept, and primer sequences were trimmed using Cutadapt v.1.13 (87). After the removal of single sequence reads, the average number of amplicon reads from the four samples was 127,515 (standard deviations [SD], 46,906). Rarefaction curves were generated for each sample using custom-made R scripts (https://github.com/khoadley/Curacao). Additionally, alpha diversity within each sample was characterized using the Shannon diversity index and reported in Table S1 in the supplemental material. For the assessment of the heterotrophic bacterial community, sequences (289,975 reads) were resolved into 2,300 amplicon sequence variants (ASVs) with USEARCH v10.0.240 (88). Taxonomies were assigned to each ASV using classify-sklearn by QIIME2 (89), searching against SILVA database release 132 (90).

Cyanobacterial and plastid amplicons were initially parsed using the phylogenetic pipeline in PhyloAssigner version v6.166 (91). Amplicons from plastids and cyanobacteria were further classified using a global plastid and cyanobacterial reference alignment and tree according to protocols outlined previously (84, 92).

### Growth, disappearance, and carbon calculations.

For each picoplankton group, growth rates between sampling intervals were utilized to assess statistical differences between the microbial groups enumerated by flow cytometry in controls and coral treatments. First, growth rates for each time interval were calculated from controls as growth rates (μ) at 1 h = log(T_1_/*T*_0_) and μ at 5 h = log(*T*_5_/T_1_), where *T*_0_, *T*_1_, and *T*_5_ represent the numeric cell abundance for each microbial group at initial, 1-h, and 5-h time points, respectively. For simplicity, growth rates over the full 5-h incubation were also calculated as μ 5 h = log(*T*_5_/*T*_0_), and the minimum daily growth rate was taken as the sum of the postsunset and presunrise growth rates in controls. All of these calculations were performed for each treatment flask as well.

### Carbon estimates.

For estimating carbon cell^−1^, the values 39 fg cell^−1^ (*Prochlorococcus*), 82 fg cell^−1^ (*Synechococcus*), 530 fg cell^−1^ (photosynthetic eukaryotes), and 20 fg carbon cell^−1^ (heterotrophic bacteria) were used, based on CHN calibrated values from natural populations (41, 42). Carbon estimates were then utilized to calculate total microbial carbon within the water column, minimum daily carbon produced, and the quantity of carbon removed from the water column by coral predation. Carbon removal by coral predation was calculated using the total number of cells removed from the water column per hour and accounts for changes in cell abundance due to natural cell division. Natural cell growth rates (Table 1) were computed from control chambers, and for estimating carbon removal by coral predation, only colonies from the postsunset incubation period were used. Importantly, carbon removal is typically normalized to coral surface area. While this was not available for our study, all fragments were glued to 25-cm^2^ PVC tiles, which generally reflected a surface area similar to or slightly larger than that of the coral fragment. Thus, rates of carbon removal can be viewed as approximate rates for 25 cm^2^ of coral surface area. For cell abundance, carbon, and 16S amplicon data, values shown represent the averages ± SD (*n* = 4).

### Statistical analyses.

A total of six microbial groups (small photosynthetic eukaryotes, *Prochlorococcus*, three groups of *Synechococcus*, and heterotrophic bacteria) were analyzed by flow cytometry. For each, a linear mixed-model analysis with repeated measures was utilized to test for significant differences in growth rates between control and coral samples. Assumptions of normality were tested using a Shapiro-Wilk test. Samples from early and late incubation periods were analyzed separately. Cell abundance, estimated total carbon, growth rates, FALS, and red and orange fluorescence values were also statistically compared across postsunset and presunrise incubation periods using a *t* test (Table 1). For these postsunset and presunrise incubation comparisons, data for FALS and red and orange fluorescence was derived from control samples at the *T*_0_ time point. All analyses were conducted in R using the lmer package (93).

### Data availability.

Resulting 16S rRNA gene amplicon sequence reads were deposited in the SRA database (NCBI Sequence Read Archive no. PRJNA638889). R scripts for analyzing and generating figures from flow cytometry analysis are available via Github (https://github.com/khoadley/Curacao).

## Supplementary Material

Supplemental file 2

Supplemental file 1
