# Supplementary material for: Selective Uptake of Pelagic Microbial Community Members by Caribbean Reef Corals
Source: Appl Environ Microbiol. 2021 Apr 13;87(9):e03175-20. doi: 10.1128/AEM.03175-20 (PMC8091028; doi:10.1128/AEM.03175-20)
Supplement: Supplemental file 1 [file AEM.03175-20-s0001.pdf]

Supplemental Information

SI-Figure 1: Clearance Rate Rig: Picture of flow-through water bath utilized to carry out clearance rate work. Circulating seawater was pumped in from the reef and maintained corals at temperatures experience on their native reef. A total of 8 500 mL chambers (glass flasks) were utilized during each 5-hr incubation. Digital probe (center front) monitored temperature throughout all incubations.

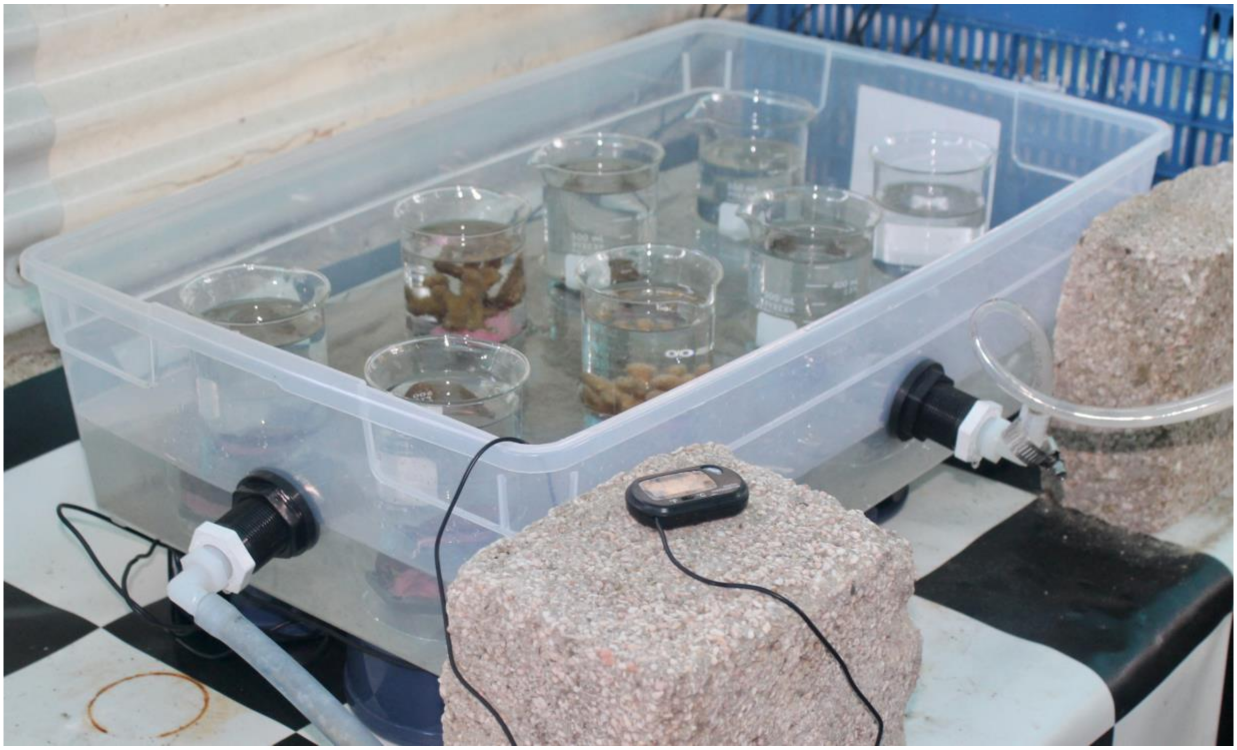

SI-Figure 2: Rarefaction Curves. Based on all ASVs analyzed within each representative seawater sample collected from the initial batch of water used to setup each incubation experiment. For each of the four curves, the number of sample amplicons post-QC is reflected by the **x-axis** whereas the number of ASVs is on the **y-axis**. Rarefaction analysis was run in R using custom scripts.

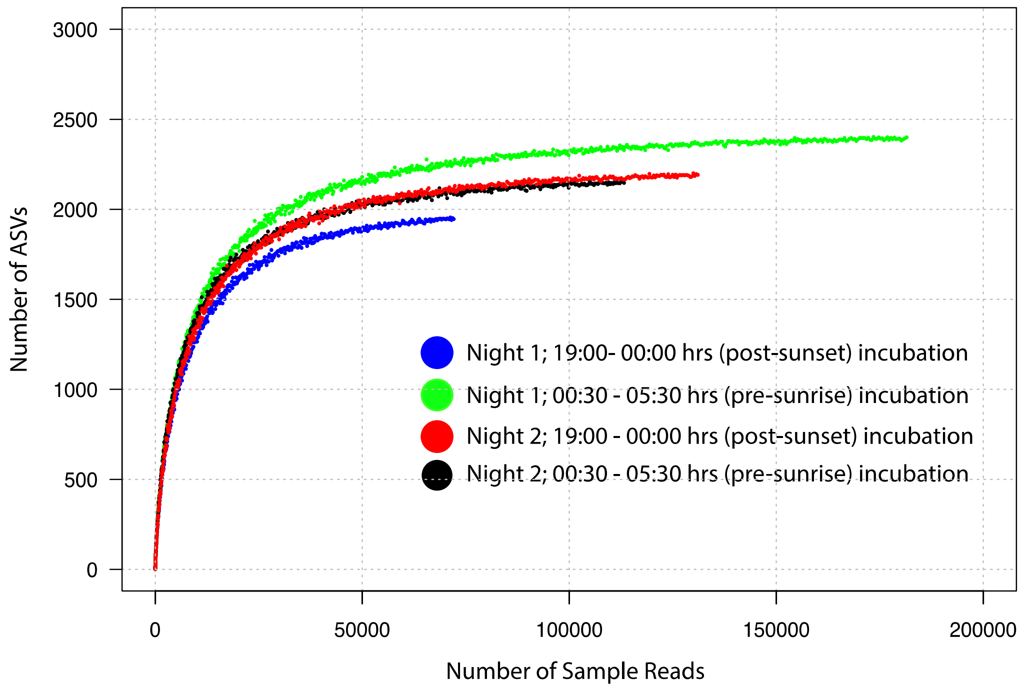

SI - Table 1: Alpha Diversity analyses. All analyses were performed in R, using custom scripts.

$$\text{Shannon diversity index (H)} = \sum[(Pi)\ x \ln(Pi)]$$

Where *Pi* equals the proportion of total sample represented by each individual ASV

$$\text{Evenness } (E_H) = \frac{H}{\ln(S)}$$

Where *S* indicates species richness or the number of ASVs and *H* is the Shannon diversity index.

**Incubation**	**Species richness (ASVs)**	**Shannon diversity index (*H*)**	**Evenness (E_H)**
Night 1, post-sunset	1995	5.76	0.76
Night 1, pre-sunrise	2486	6.01	0.77
Night 2, post-sunset	2101	5.89	0.77
Night 2, pre-sunrise	2276	5.73	0.74
Average	2214 ± 215	5.85 ± 0.13	0.76 ± 0.01

SI-Table 2: Temperature and Nutrient conditions. Temperature and nutrient samples were collected from the initial batch water utilized for each incubation. Seawater utilized within the experiment was derived from the same native reef where corals were collected. Nutrient samples were analysed via auto-analyzer and according to methods described in (Pennington and Chavez, 2000).

Incubation	Temperature (°C)	PO_4^{-3} (μM)	NO_3^- (μM)	NO_2^- (μM)
Night 1, post-sunset	27.6	0.049	0.352	0.085
Night 1, pre-sunrise	27.6	0.129	0.190	0.094
Night 2, post-sunset	27.5	0.184	0.084	0.053
Night 2, pre-sunrise	27.4	0.064	0.229	0.073

SI-Table 3: Cell Physiology during post-sunset incubation. Average FALS and red fluorescence are displayed in bead relative units (BRU) and were only calculated for *Prochlorococcus* and *Synechococcus* groups. FALS and red fluorescence values were not calculated for photosynthetic eukaryotes as they reflect a much more biologically diverse assemblage and the averages would not be informative. Average orange fluorescence (BRU) was only calculated for *Synechococcus* as they contain phycoerythrin. Bold values reflect significant differences (T-test, *p*-value shown) across incubation periods.

**Microbial Group**	**Post-sunset Incubation (T_0)**	**Post-sunset Incubation (T_5)**	***P*-value**
	FALS (BRU)	FALS (BRU)	
Prochlorococcus	**0.45 ± 0.02**	**0.30 ± 0.02**	**0.0001**
***Synechococcus* group 1**	**0.80 ± 0.02**	**0.69 ± 0.02**	**0.0003**
***Synechococcus* group 2**	**0.68 ± 0.04**	**0.55 ± 0.03**	**0.0016**
***Synechococcus* group 3**	**0.69 ± 0.02**	**0.64 ± 0.03**	**0.0452**
	Red Fluor (BRU)	Red Fluor (BRU)	
Prochlorococcus	0.77 ± 0.04	0.61 ± 0.03	0.3051
***Synechococcus* group 1**	**2.19 ± 0.07**	**2.03 ± 0.10**	**0.0025**
Synechococcus group 2	1.75 ± 0.04	1.61 ± 0.07	0.1009
Synechococcus group 3	1.57 ± 0.02	1.55 ± 0.02	0.5728
	Orange Fluor (BRU)	Orange Fluor (BRU)	
***Synechococcus* group 1**	**1.40 ± 0.03**	**1.29 ± 0.02**	**0.0397**
***Synechococcus* group 2**	**0.99 ± 0.09**	**0.88 ± 0.05**	**0.0270**
Synechococcus group 3	0.73 ± 0.09	0.70 ± 0.06	0.2203

SI-Table 4: Cell Physiology during pre-sunrise incubation. Average FALS and red fluorescence are displayed in bead relative units (BRU) and were only calculated for *Prochlorococcus* and *Synechococcus* groups. FALS and red fluorescence values were not calculated for photosynthetic eukaryotes as they reflect a much more biologically diverse assemblage and the averages would not be informative. Average orange fluorescence (BRU) was only calculated for *Synechococcus* as they contain phycoerythrin. Bold values reflect significant differences (T-test, *p*-value shown) across incubation periods.

**Microbial Group**	**Pre-sunrise Incubation (T_0)**	**Pre-sunrise Incubation (T_5)**	***P*-value**
	FALS (BRU)	FALS (BRU)	
Prochlorococcus	0.30 ± 0.01	0.29 ± 0.00	0.3171
Synechococcus group 1	0.70 ± 0.00	0.70 ± 0.01	0.3851
Synechococcus group 2	0.58 ± 0.02	0.59 ± 0.04	0.8056
Synechococcus group 3	0.68 ± 0.03	0.68 ± 0.03	0.8773
	Red Fluor (BRU)	Red Fluor (BRU)	
Prochlorococcus	0.69 ± 0.08	0.71 ± 0.07	0.3237
Synechococcus group 1	2.02 ± 0.06	1.99 ± 0.11	0.7182
Synechococcus group 2	1.69 ± 0.13	1.68 ± 0.21	0.8782
Synechococcus group 3	1.56 ± 0.09	1.57 ± 0.11	0.8045
	Orange Fluor (BRU)	Orange Fluor (BRU)	
***Synechococcus* group 1**	1.35 ± 0.07	1.31 ± 0.13	**0.7332**
Synechococcus group 2	1.01 ± 0.13	0.98 ± 0.23	0.9431
Synechococcus group 3	0.80 ± 0.09	0.77 ± 0.17	0.9569

SI-Table 5: Carbon quantity filtered out of the water column hr^{-1} per coral colony. All coral colonies were trimmed to be similar in size (~25-cm^2 surface area), but values were not normalized to coral surface area and are instead reported per coral colony.

	Coral	*M. madracis*	*P. astreoides*	*S. intersepta*
Prochlorococcus sp.	C (ng hr^{-1})	215.7 ± 103.6	42.8 ± 35.6	94.7 ± 54.5
Synechococcus sp.	C (ng hr^{-1})	70.7 ± 58.7	9.2 ± 4.7	20.1 ± 12.2
Eukaryotes	C (ng hr^{-1})	101.6 ± 18.2	65.5 ± 6.9	47.5 ± 38.5
**Total Carbon**	**C (ng hr^{-1})**	**387.9 ± 152.6**	**106.8 ± 27.3**	**138.7 ± 62.2**

References:

Pennington JT, Chavez FP. Seasonal fluctuations of temperature, salinity, nitrate, chlorophyll and primary production at station H3/M1 over 1989–1996 in Monterey Bay, California, Deep Sea Research Part II: Topical Studies in Oceanography. 2000;47:947-973.
